# Genome wide characterization of enterotoxigenic *Escherichia coli* serogroup O6 isolates from multiple outbreaks and sporadic infections from 1975-2016

**DOI:** 10.1371/journal.pone.0208735

**Published:** 2018-12-31

**Authors:** Vaishnavi Pattabiraman, Lee S. Katz, Jessica C. Chen, Andre E. McCullough, Eija Trees

**Affiliations:** 1 Centers for Disease Control and Prevention, Atlanta, GA, United States of America; 2 IHRC Inc, Atlanta, GA, United States of America; 3 Center for Food Safety, College of Agricultural and Environmental Sciences, University of Georgia, Griffin, GA, United States of America; Laboratoire National de Santé, LUXEMBOURG

## Abstract

Enterotoxigenic *Escherichia coli* (ETEC) are an important cause of diarrhea globally, particularly among children under the age of five in developing countries. ETEC O6 is the most common ETEC serogroup, yet the genome wide population structure of isolates of this serogroup is yet to be determined. In this study, we have characterized 40 ETEC O6 isolates collected between 1975–2016 by whole genome sequencing (WGS) and by phenotypic antimicrobial susceptibility testing. To determine the relatedness of isolates, we evaluated two methods—whole genome high-quality single nucleotide polymorphism (whole genome-hqSNP) and core genome SNP analyses using Lyve-SET and Parsnp respectively. All isolates were tested for antimicrobial susceptibility using a panel of 14 antibiotics. ResFinder 2.1 and a custom quinolone resistance determinants workflow were used for resistance determinant detection. VirulenceFinder 1.5 was used for prediction of the virulence genes. Thirty-seven isolates clustered into three major clades (I, II, III) by whole genome-hqSNP and core genome SNP analyses, while three isolates included in the whole genome-hqSNP analysis only did not cluster with clades I-III by both analyses and formed a distantly related outgroup, designated clade IV. Median number of pairwise whole genome-hqSNPs in clonal ETEC O6 outbreaks ranged from 0 to 5. Of the 40 isolates tested for antimicrobial susceptibility, 18 isolates were pansusceptible. Twenty-two isolates were resistant to at least one antibiotic, nine of which were multidrug resistant. Phenotypic antimicrobial resistance (AR) correlated with AR determinants in 22 isolates. Thirty-two isolates harbored both enterotoxin virulence genes while the remaining 8 isolates had only one of the two virulence genes. In summary, whole genome-hqSNP and core genome SNP analyses from this study revealed similar evolutionary relationships and an overall diversity of ETEC O6 isolates independent of time of isolation. Less than 5 pairwise hqSNPs between ETEC O6 isolates is circumstantially indicative of an outbreak cluster. Findings from this study will be a basis for quicker outbreak detection and control by efficient subtyping by WGS.

## Introduction

Enterotoxigenic *Escherichia coli* (ETEC) is the leading bacterial cause of foodborne illnesses worldwide [1] and is the most common cause of bacterial diarrhea in children under the age of five in low and middle-income countries and in travelers to endemic areas [2, 3]. ETEC strains secrete heat-labile (LT) and/or heat-stable (ST) enterotoxins and produce colonization factors, which are fimbrial, afimbrial or fibrillar surface structures that mediate adherence to the human intestinal mucosa. For disease presentation, the enterotoxins must be successfully delivered to cognate receptors on epithelial cells of the small intestine, where ensuing loss of water and electrolytes results in diarrhea [4]. ETEC strains are genetically diverse and over a 100 different O antigens have been associated with clinical ETEC isolates [5–7] with O6 being the most common serogroup, both in the frequency of isolates and the number of geographic locations from where it was recovered [6, 8]. Both LT and ST have been reported in O6 strains [9, 10].

In upper-middle income countries such as Peru, fluoroquinolones (ciprofloxacin or norfloxacin) and azithromycin remain the drugs of choice for ETEC infection [11] whereas in lower-income countries such as Bangladesh high levels of antimicrobial resistance (>40%) to multiple individual antibiotics have been observed in addition to resistance to treatment options such as fluoroquinolones [12]. Centers for Disease Control and Prevention (CDC) indicate that fluoroquinolones are shown to be an effective therapy in ETEC mediated traveler’s diarrhea [13]. Collectively, these studies connote that while fluoroquinolones remain the most common drug of choice, effective antibiotic treatment for ETEC mediated diarrhea may vary slightly depending on the geographical location.

A few comparative genomics studies of ETEC have been conducted that have shown substantial genetic diversity in this pathotype [10, 14–16]. For instance, von Mentzer *et al* [14] used next-generation sequencing (NGS) for a complete understanding of ETEC phylogeny and evolution using a global collection of ETEC isolates collected between 1980 and 2011 while Sahl *et al* [15] characterized phylogenomic diversity through the identification of genetically distinct pathogenic isolates within an ETEC infected individual by genome sequencing and comparative analysis.

However, no genome wide population studies were conducted on ETEC O6 isolates which is the most common ETEC serogoup. Here, we use NGS and antimicrobial susceptibility testing to characterize ETEC O6 isolates from multiple outbreaks and sporadic infections from 1975–2016. We describe the phylogeny and evolution of O6 isolates by whole genome high quality single nucleotide polymorphism (WG-hqSNP) and core genome SNP (CG SNP) analyses. We document the nature of antimicrobial resistance by phenotypic and genotypic antimicrobial resistance determination in these isolates. Understanding the genetic structure of the population as well as variation among strains that are likely to be epidemiologically related or unrelated will be helpful as many surveillance networks, including PulseNet USA, the molecular network for foodborne disease surveillance, moves forward with implementation of whole genome sequencing (WGS) and establishment of interpretive guidance.

## Materials and methods

### Bacterial strains

To characterize historic and recent ETEC serogroup O6 strains by WGS we selected 40 ETEC O6 isolates [38 O6:H16; 2 O6: non-motile (NM)] from 1975–2016 for inclusion in our study. At least one isolate was selected from each of the 18 outbreaks and 13 sporadic infections for further characterization. The isolates belonged to the Centers for Disease Control and Prevention (Atlanta, GA) isolate collection. ETEC O6:H16 serotypes were predicted by SerotypeFinder-1.1 in the Center for Genomic Epidemiology (CGE) [17] website while O6:NM serotypes were confirmed by standard serotyping laboratory procedure [18]. These isolates are listed in S1 Table.

### Total DNA extraction and quantification

Total DNA were extracted from the ETEC O6 isolates (S1 Table) using the DNeasy Blood and Tissue Kit (Qiagen Inc) as described in the Laboratory Standard Operating Procedure (SOP) for PulseNet Nextera XT library preparation and run set up for Illumina MiSeq [19]. Purity of DNA at 260/280 nm was confirmed by Nanodrop 2000 spectrophotometer (Thermo Scientific) and concentration of extracted DNA was determined by a Qubit 2.0 fluorometer (Life Technologies) using dsDNA BR assay kit. DNA that passed the quality control with a purity of >/ = 1.75 at 260/280 nm and with a minimum concentration of 10 ng/μl were subjected to NGS by MiSeq (Illumina Inc, San Diego, CA).

### Whole genome sequencing

Sequencing libraries for 40 ETEC O6 isolates (S1 Table) were prepared by Nextera XT DNA library preparation kit as described [19]. Denatured pooled libraries were loaded at a final concentration of 10 pM on the flow cell. 500-cycle chemistry (2 x 250 bp reads) was used for sequencing the genomes of all isolates in this study. The PulseNet-recommended minimum quality thresholds of ≥ 40x coverage and average Q30 score ≥ 30 were followed for all sequenced genomes in this study [20]. The genome of the reference isolate 2011EL1370-2 (S1 Table) was sequenced using PacBio (Pacific Biosciences, Menlo Park, CA) as previously described [21] and the sequence reads were filtered and assembled *de novo* using the PacBio Hierarchical Genome Assembly [22] to yield 1 contiguous DNA sequence for the chromosome and two contiguous DNA sequences for two plasmids.

### Bioinformatics analyses

Raw sequence reads were subjected to quality control using PRINSEQ version 0.20.3 [23] with the following metrics: 22 bases from the 5’end and 10 bases from the 3’end were trimmed, in order to reach a minimum mean quality score of 25. Lyve-SET v1.1.4f [24] is a hqSNP pipeline designed to identify SNPs with high quality while removing lower quality SNPs from its analysis, resulting in a high-confidence phylogeny. Reads passing quality control were used as input for the Lyve-SET and *de-novo* genome assembly by SPAdes genome assembler v3.9.0 [25]. Lyve-SET was applied using the reference genome with the following conditions: phages in the reference genome were masked, clustered SNPs within 5 bp were filtered, SNPs calls had to be supported by at least 20x coverage and 95% consensus. Phylogenetic trees inferred by Randomized Axelerated Maximum Likelihood (RAxML) tool [26] integrated in the Lyve-SET pipeline were viewed and formatted in MEGA6 [27]. Phylogenetic trees were midpoint rooted and the taxa were formatted for balanced shape. Lyve-SET also generates a pairwise matrix output file listing the number of pairwise hqSNPs between the genomes in this study. SPAdes was run using the following conditions:—careful (to reduce the number of mismatches and short indels) and—only-assembler (runs assembly module only).

We performed CG-SNP analysis of all the genomes against the reference genome using Parsnp version 1.2, a rapid conservative core genome multi-alignment tool, using default parameters [28]. By default, Parsnp calculates the Maximal Unique Matches Index (MUMi) distances between the reference and each of the genomes in the genome library. MUMi is a genomic distance based on the amount of exact matches at least 19 bp long shared between two genomes [29]. It only includes genomes with MUMi distance < = 0.01 and excludes genomes that are outside of this parameter. We used the assembled genomes of all ETEC O6 isolates in the genome directory as input for CG alignment by Parsnp. Parsnp yielded a phylogenetic tree that was then viewed and formatted in MEGA6.

We compared the trees resulting from Parsnp and Lyve-SET using a method that we previously developed [24]. Briefly, 10,000 trees with random topologies were created for each comparison, with either the Parsnp tree or the Lyve-SET tree designated as the reference or the query tree. The observed Robinson-Foulds values were calculated, and the average values for each metric were recorded between the random trees and the reference tree. We compared the observed and background distribution with the Z-test to determine how significantly different the observed tree distance was between trees. Next, we approximated the difference in branch length of Clade II in CG-SNP and WG-hqSNP phylogenies by subtraction of the SNPs between the two phylogenies.

We searched the National Center for Biotechnology Information (NCBI) Pathogen Detection Pipeline (https://www.ncbi.nlm.nih.gov/pathogens) for any related genomes to the set of genomes in clade IV (S1 Fig). The Pathogen Detection Pipeline organizes all publicly available genomes on NCBI that are closely related into trees based on the Jaccard distance and these genomes are refined into subclades using SNPs found among related assemblies [24].

### Antimicrobial susceptibility testing

Antimicrobial susceptibility testing (AST) was performed according to standard NARMS methodology using broth microdilution on the Sensititre Gram-negative susceptibility panel CMV4AGNF (Thermo Scientific, Inc) [30].

Inoculum was prepared by generating a 0.5 McFarland suspension in 5ml sterile demineralized water for each isolate. A 10μl aliquot of water inoculum was then transferred to 11ml of cation adjusted Mueller Hinton Broth. A 50μl aliquot of broth inoculum suspension was transferred into each well of the 96-well Gram-negative susceptibility panel using the Sensititre AIM automated inoculation delivery system. Panels were incubated at 35°C for 18–20 hours. Results were read using the automated reading and incubation system (ARIS, Thermo Scientific Inc).

The Gram negative panel was comprised of 14 antimicrobial agents and the interpretive criteria for being susceptible or resistant to a drug was followed as previously described [30]. The criteria used to categorize minimum inhibitory concentration (MIC) results as susceptible, intermediate or resistant are based on current guidelines provided by the Clinical Laboratory Standards Institute (CLSI) where available or based on NARMS consensus epidemiologic cutoffs (for streptomycin) [30, 31]. A pansusceptible isolate is defined as an isolate susceptible to the Gram negative panel of 14 antimicrobial agents while a multidrug resistant isolate is defined as resistance to 3 or more CLSI drug classes [31].

### Identification of antimicrobial resistance determinants

Assembled genomes of all the isolates in this study (S1 Table) were input into the ResFinder 2.1 tool in the CGE website (https://cge.cbs.dtu.dk) for identification of acquired AR genes, in August 2017. Default thresholds of 90% identity and 60% gene coverage were employed [30, 32]. Additional variants of the *mcr* and *qnr* mobile resistance determinants that confer resistance to polymyxin (e.g. colistin) and fluoroquinolone (e.g. ciprofloxacin) classes of antibiotics [33, 34] were discovered after the initial screening. Determinants present on contigs with low sequencing coverage (defined as less than 5x) were omitted from further analysis as these are sometimes the result of carryover contamination or sample bleed over on the Illumina platform [35]. To this end, we ran a MegaBLAST using NCBI-blast+ with a 90% identity cutoff to detect *mcr-3*, *mcr-4 and qnrE* determinants in the assembled genomes while the other variants of these genes were screened by ResFinder 2.1.We used an in-house script to extract sequences encoding DNA gyrase (*gyrA*, *gyrB)* and topoisomerase IV (*parC*) (https://github.com/lskatz/ETEC-O6) from the assembled genomes. Multiple sequence alignment in MEGA6 by ClustalW of the translated DNA sequence was used for the detection of mutations with particular emphasis on mutations known to confer resistance in the quinolone resistance determining regions (QRDRs) of these genes [e.g. mutations occurring at *gyrA*(S^83^), *gyrA*(D^87^), *parC*(S^80^)] [36–38].

Phenotypic antimicrobial resistance and resistance determinants were visualized alongside WG-hqSNP phylogeny using the ggtree package in R v. 3.5.1 [39].

### Identification of virulence factors

Assembled genomes of all the isolates in this study (S1 Table) were input into the VirulenceFinder 1.5 tool in the CGE website (https://cge.cbs.dtu.dk) for identification of enterotoxin virulence genes [40].

## Results

### Whole genome high-quality SNP phylogeny of ETEC O6 isolates

We analyzed a total of 40 ETEC O6 isolates, 27 of which were from 18 outbreaks and 13 from sporadic cases spanning from 1975–2016 collected from Central America, USA and Guatemala (S1 Table). We employed WG-hqSNP analysis by Lyve-SET to construct the WG phylogeny of the 40 ETEC O6 isolates. The three clade IV genomes were 2,840–7,657 pairwise hqSNPs different from the other genomes included in this study, indicating that these three genomes were an outgroup (S1 Fig) and hence too diverse to be included in the further phylogenetic analyses.

**Fig 1:**
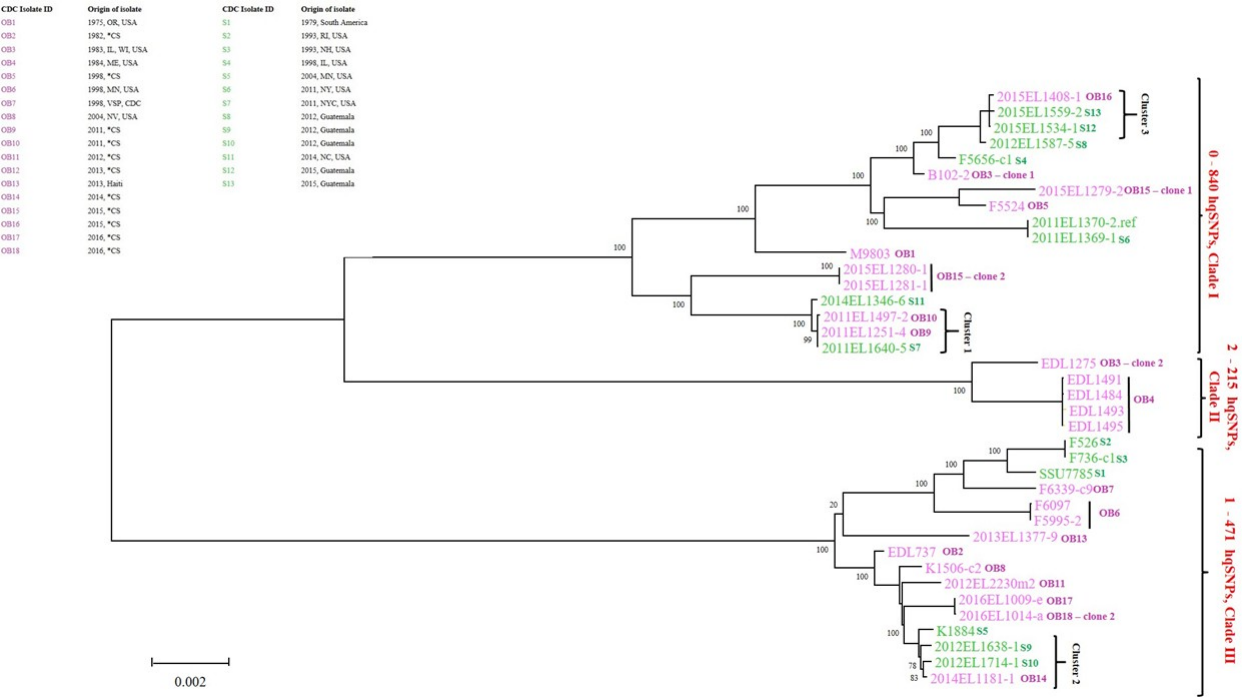
ETEC O6 isolates are clustered into three clades by whole genome high-quality SNP analysis. Whole genome high-quality single nucleotide polymorphisms (WG-hqSNP) tree generated by Lyve-SET for phylogenetic relatedness of 37 ETEC O6 isolates against the reference genome 2011EL1370-2. The scale represents a distance of 0.002 hqSNPs per site. At each ancestor node, bootstrap percentages are displayed. Isolates are clustered into Clade I, Clade II and Clade III. Isolates are color coded based on isolation during an outbreak (OB) or sporadic infection (S). In the metadata table, *CS stands for Cruise Ship; US states are abbreviated.

Results from WG-hqSNP analysis indicated that ETEC O6 isolates clustered into three major clades (I, II and III) (Fig 1). Clade I comprised of 9 outbreak and 8 sporadic isolates, clade II comprised of 5 outbreak isolates and clade III comprised of 10 outbreak and 6 sporadic isolates. Clade I isolates differed by 0–840 pairwise hqSNPs with a median of 372 pairwise hqSNPs, clade II isolates differed by 2–215 pairwise hqSNPs with a median of 7 pairwise hqSNPs and clade III isolates differed by 1–471 pairwise hqSNPs with a median of 177 pairwise hqSNPs.

### Core genome SNP phylogeny of ETEC O6 isolates

**Fig 2:**
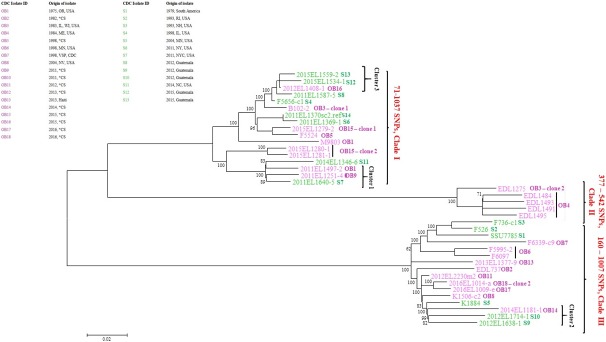
ETEC O6 isolates are clustered into three clades by core genome SNP analysis. Core genome single nucleotide polymorphisms (CG-SNP) tree generated by Parsnp for phylogenetic relatedness of 37 ETEC O6 isolates against the reference genome 2011EL1370-2. The scale represents a distance of 0.02 SNPs per site. Bootstrap percentages are displayed at each ancestor node. Isolates are clustered into Clade I, Clade II and Clade III. Isolates are color coded based on isolation during an outbreak (OB) or sporadic infection (S). In the metadata table, *CS stands for Cruise Ship; US states are abbreviated.

ETEC O6 genomes clustered into three major clades (I, II and III) by CG-SNP analysis performed using Parsnp (Fig 2). Each of the three clades consisted of the same outbreak and sporadic infection isolates as seen in the phylogeny by WG-hqSNP analysis (Fig 1). Clade I genomes differed by 71–1037 pairwise SNPs with a median of 703 pairwise SNPs, clade II genomes differed by 377–542 pairwise SNPs with a median of 423 pairwise SNPs and clade III genomes differed by 160–1007 pairwise SNPs with a median of 601 pairwise SNPs (Fig 2). By default Parsnp excluded the three outgroup genomes (clade IV, S1 Fig) in its analysis because the MUMi distances of these genomes were > 0.01.

### Comparison of whole genome high-quality SNP and core genome SNP phylogenies

Overall, the WG-hqSNP and CG SNP phylogenies of ETEC O6 isolates in this study were similar (Figs 1 and 2). When comparing the topologies of both trees, we found that they are more similar than what we would expect by chance alone (Robinson-Foulds metric of 30, *p* < 1e-99). However, the branch length of clade II in CG-SNP phylogeny was longer when compared to the branch length of clade II in WG-hqSNP phylogeny. We further investigated and attributed the difference in this branch length to 871 pairwise SNPs present in the CG-SNP phylogeny that were not detected in WG-hqSNP phylogeny.

### Median number of whole genome high-quality SNPs in ETEC O6 isolates associated with outbreaks

Detecting clusters of isolates from cases that could be associated with a common source using WGS data requires an understanding of within cluster genome diversity for the establishment of approximate SNP or allele cut-off thresholds. A clonal outbreak is caused by a single strain while a polyclonal outbreak is caused by multiple genetically different strains of the same serotype, multiple serotypes of the same species or multiple species. With the underlying goal of ascertaining clonal or polyclonal ETEC O6 outbreaks in the future based on the pairwise hqSNP counts, we next determined the median number of pairwise hqSNPs in genomes of ETEC O6 isolates belonging to epidemiologically confirmed outbreaks. The median number of pairwise hqSNPs in OB4, OB6, OB9, OB10 and OB12 outbreaks were between 0–5 implying that these were clonal outbreaks (Table 1). In OB15, median number of pairwise hqSNPs in clone 2 was 0 while 759–835 pairwise hqSNPs were detected between isolates belonging to clones 1 and 2 insinuating that OB15 was a polyclonal outbreak. In total, 1,908 pairwise hqSNPS were detected between isolates belonging to clones 1 and 2 of OB3 suggesting that this was also a polyclonal outbreak. Median number of pairwise hqSNPs for OB17 and clone 1 of OB18 was 1 while 5,436 pairwise hqSNPs were identified between clones 1 and 2 of OB18 indicating polyclonality. Overall, based on this study results the median number of pairwise hqSNPs in clonal ETEC O6 outbreaks was less than 5.

**Table 1:** Median number of pairwise whole genome high-quality SNPs in ETEC O6 outbreaks are 0–4.5.

Outbreak	Number of isolates	Median number of hqSNPs ^1^	Predicted outbreak type
OB3 –clone 1	1	-	Polyclonal
OB3 –clone 2	1	-	Polyclonal
OB4	4	4.5	Clonal
OB6	2	1	Clonal
OB9, OB10	2	4	Clonal
OB12	2	0	Clonal
OB15 –clone 1	1	-	Polyclonal
OB15 –clone 2	2	0	Polyclonal
OB17, OB18-clone 1	2	1	Clonal
OB18 –clone 2	1	-	Polyclonal

OB stands for outbreak. ^1^A dash in this column indicates that only one isolate for this clone was available for study and therefore the median could not be calculated.

Few sporadic infections isolates in clades I and III have clustered closely with outbreak isolates in three clusters where the median number of pairwise hqSNPs were between 4–14 (Table 2).

**Table 2:** Median number of pairwise whole genome high-quality SNPs in clusters of isolates from sporadic infections and outbreaks.

Cluster number	CDC isolate ID (sporadic infection/outbreak number)	Median number of pairwise WG-hqSNPs
1 2 3	2011EL1640-5 (S7), 2011EL1251-4 (OB 9), 2011EL1497-2 (OB10) 2012EL1714-1 (S8), 2012EL1638-1 (S8), 2014EL1181-1 (OB14) 2015EL1534-1 (S10), 2015EL1559-2 (S10), 2015EL1408-1 (OB16)	4 14 9

OB stands for outbreak; S stands for sporadic infection.

OB stands for outbreak; S stands for sporadic infection.

#### Antimicrobial resistance

**Fig 3:**
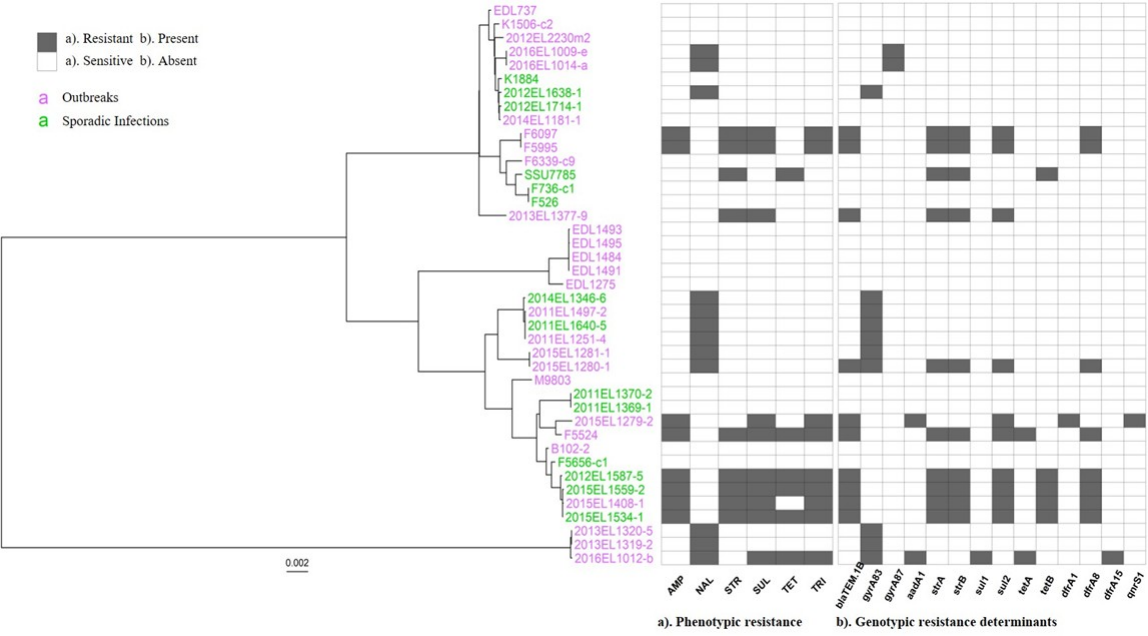
Heat map visualization of phenotypic and genotypic antimicrobial resistance patterns of ETEC O6 isolates. Phenotypic antimicrobial resistance and resistance determinants were visualized alongside WG-hqSNP phylogeny using the ggtree package in R. a). Phenotypic antimicrobial resistance patterns: AMP: Ampicillin; NAL: Nalidixic acid; STR: Streptomycin; SUL: Sulfisoxazole; TET: Tetracycline; TRI: trimethoprim-sulfamethoxazole. b). Genotypic resistance determinants: *bla*_TEM-1B_: beta-lactam resistance determinant; *gyrA83*, *gyrA87*: point mutations in S83L and D87Y in Gyrase A subunit of DNA Gyrase enzyme resulting in reduced binding of nalidixic acid; *aadA1*, *strA*, *strB*: streptomycin resistant determinants; *sul1*, *sul2*: sulphonamide resistance determinant; *tetA*, *tetB*: tetracycline resistance determinant; *dfrA1*, *dfrA8* and *dfrA15*: trimethoprim-sulfamethoxazole resistance determinant; *qnrS1*: quinolone resistance determinant.

All isolates in the study were analyzed for antimicrobial susceptibility. Eighteen ETEC O6 isolates were pansusceptible, and the remaining 22 isolates were resistant to one or more antibiotics (Table 3). Heat map visualization of phenotypic resistance and genotypic resistance determinants are shown (Fig 3). Out of the resistant isolates, nine were multidrug resistant. Among the resistant isolates, phenotypic resistance was observed to one or more of the following drugs: ampicillin, nalidixic acid, streptomycin, sulfonamides, tetracycline, trimethoprim-sulfamethoxazole. All isolates were susceptible to amoxicillin-clavulanic acid, azithromycin, cefoxitin, ceftriaxone, chloramphenicol, ciprofloxacin, gentamicin and meropenem. Resistance was correlated in 22 isolates with one or more of the following genotypic AR determinants: *bla*_TEM-1B_, *aadA1*, *strA*, *strB*, *sul1*, *sul2*, *tetA*, *tetB*, *dfrA1*, *dfrA8*, *dfrA15*, and mutations in *gyrA*. The *qnrS1* gene was detected in one isolate lacking phenotypic resistance to quinolones. No known *mcr* mediated colistin resistance determinants were found.

**Table 3:** Phenotypic and genotypic antimicrobial resistance profiles of ETEC O6 isolates.

CDC Isolate ID	Outbreak (OB)/Sporadic (S) infection number	Phenotypic resistance resistance profile	Resistance determinants
F5524	OB5	A,S,Su,T,Cot	*bla*_TEM-1B_, *strA*, *strB*, *sul2*, *tetA*, *dfrA8*
F5995	OB6	A,S,Su,Cot	*bla*_TEM-1B_, *strA*, *strB*, *sul2*, *dfrA8*
F6097	OB6	A,S,Su,Cot	*bla*_TEM-1B_, *strA*, *strB*, *sul2*, *dfrA8*
2011EL1251-4	OB9	Nal	*gyrA83*
2011EL1497-2	OB10	Nal	*gyrA83*
2013EL1319-2	OB12	Nal	*gyrA83*
2013EL1320-5	OB12	Nal	*gyrA83*
2013EL1377-9	OB13	S,Su	*bla*_TEM-1B_, *strA*, *strB*, *sul2*
2015EL1279-2	OB15	A,Su,Cot	*bla*_TEM-1B_, *aadA1*, *sul2*, *dfrA1*, *qnrS1*
2015EL1280-1	OB15	Nal	*bla*_TEM-1B_, *gyrA83*, *strA*, *strB*, *sul2*, *dfrA8*
2015EL1281-1	OB15	Nal	*gyrA83*
2015EL1408-1	OB16	A,S,Su,Cot	*bla*_TEM-1B,_ *strA*, *strB*, *sul2*, *tetB*, *dfrA8*
2016EL1009-e	OB17	Nal	*gyrA87*
2016EL1012-b	OB18	Nal,Su,T,Cot	*gyrA83*, *aadA1*, *sul1*, *tetA*, *dfrA15*
2016EL1014-a	OB18	Nal	*gyrA87*
SSU7785	S1	S,T	*strA*, *strB*, *tetB*
2011EL1640-5	S7	Nal	*gyrA83*
2012EL1587-5	S8	A,S,Su,T,Cot	*bla*_TEM-1B_, *strA*, *strB*, *sul2*, *tetB*, *dfrA8*
2012EL1638-1	S9	Nal	*gyrA83*
2014EL1346-6	S11	Nal	*gyrA83*
2015EL1534-1	S12	A,S,Su,T,Cot	*bla*_TEM-1B_, *strA*, *strB*, *sul2*, *tetB*, *dfrA8*
2015EL1559-2	S13	A,S,Su,T,Cot	*bla*_TEM-1B_, *strA*, *strB*, *sul2*, *tetB*, *dfrA8*

Discrepancy between phenotypic AR and genotypic AR determinants were detected in 5 ETEC O6 isolates. 2015EL1279-2 was susceptible to streptomycin (MIC = 8 μg/mL) and ciprofloxacin (MIC = 0.25 μg/mL) while *aadA1* and *qnrS1* were detected; 2015EL1280-1 was susceptible to ampicillin (MIC = 2 μg/mL), streptomycin (MIC = 8 μg/mL), sulfisoxazole (MIC < = 16 μg/mL) and trimethoprim-sulfamethoxazole (MIC < = 0.12/2.38 μg/mL) while *bla*_TEM-1B_, *strA*, *strB*, *sul2* and *dfrA8* were detected; 2013EL1377-9 was susceptible to ampicillin (MIC = 2 μg/mL) while *bla*_TEM-1B_ was detected; 2012EL1408-1 was susceptible to tetracycline while *tetB* (MIC < = 4 μg/mL) was detected and 2016EL1012-b was susceptible to streptomycin (MIC = 16 μg/mL), yet an *aadA1* gene was present in this isolate (Table 3).

### Phenotypic resistance

A: Ampicillin; Nal: Nalidixic acid; S: Streptomycin; Su: Sulfisoxazole; T: Tetracycline; Cot: trimethoprim-sulfamethoxazole. *bla*_TEM-1B_: beta-lactam resistance determinant; *gyrA83*, *gyrA87*: point mutations in S83L and D87Y in Gyrase A subunit of DNA Gyrase enzyme resulting in reduced binding of nalidixic acid; *aadA1*, *strA*, *strB*: streptomycin resistant determinants; *sul1*, *sul2*: sulphonamide resistance determinant; *tetA*, *tetB*: tetracycline resistance determinant; *dfrA1*, *dfrA8* and *dfrA15*: trimethoprim-sulfamethoxazole resistance determinant; *qnrS1*: quinolone resistance determinant.

All isolates were susceptible to CLA: Amoxicillin-clavulanic acid, AZT: Azithromycin, CEF: Cefoxitin, CEFT: Ceftriaxone, CHL: Chloramphenicol, CIP: Ciprofloxacin, GEN: Gentamicin and MER: Meropenem.

The following isolates were pansusceptible to all antibiotics tested by antimicrobial susceptibility testing and its genomes had no antibiotic resistance determinants: K1884, F6339-c9, 2012EL2230m2, B102-2, 2014EL1181-1, 2011EL1369-1, 2012EL1714-1, F526, F736-c1, EDL1493, EDL1495, EDL1484, EDL1491, F5656-c1, EDL737, EDL1275, K1506-c2, M9803, 2011EL1370-2 (reference).

### Virulence factors

Thirty-two isolates were predicted to harbor heat-labile enterotoxin A subunit *(ltcA)* and heat-stable enterotoxin 1 *(astA)* virulence factors; 6 and 2 isolates were predicted to harbor *astA* only and *ltcA* only respectively (S1 Table).

## Discussion

Globally, O6 is the most common ETEC O serogroup [8, 14]; it is therefore important to characterize O6 strains phylogenetically to understand the evolution and genetic diversity. In this study, we performed genome wide characterization of historical and recently collected ETEC O6 isolates in order to understand the phylogenetics of this serogroup, which has a significant public health impact. Effective subtyping by WGS during future outbreaks will pave the way to quicker outbreak investigations that encompasses creation of an epidemic curve, case definition, and detection of the infection source ultimately resulting in the containment of the outbreak. This study is unique because, to our knowledge, it is the first to compare whole genome and core genome SNP analyses, determine the median number of pairwise hqSNPs in a clonal outbreak and characterize antimicrobial resistance of ETEC O6 isolates. Information gathered in this study will be part of a larger data set to be used to validate whole genome multi-locus sequence typing (wgMLST) for *E*. *coli* by PulseNet USA and PulseNet International [41].

Our results indicate that ETEC O6 isolates are genetically diverse, grouped into 3 clades by WG-hqSNP and CG SNP analyses independent of the time of isolation. WG-hqSNP identified the three outgroup genomes (clade IV, S1 Fig) that were removed from downstream WG-hqSNP and automatically excluded from CG-SNP analyses. These genomes were also diverse from other publicly available *E*. *coli* and *Shigella* genomes. Furthermore, these were confirmed as ETEC O6 by sequence based methods. As *E*. *coli* is subjected to homologous and non-homologous recombination [42], we speculate recombination to be the reason for extensive genetic diversity seen in the aforementioned genomes while selective pressure to maintain the lipopolysaccharide (LPS) component of the outer membrane (OM) of these isolates could have resulted in preservation of the O6 antigen. Phylogenetic relatedness between the O6 isolates is similar by WG-hqSNP and CG-SNP analyses indicating that these are likely to be true relationships (Figs 1 and 2). ETEC has been shown to partially cluster by phylogenetic analysis based on the O-antigen [14]. Therefore, the robustness of the phylogenetic analyses of ETEC O6 isolates by WG-hqSNP and CG-SNP analyses seen in this study will most likely not be evident if isolates of different O-serogroups were included.

The number of pairwise SNPs is consistently higher by CG-SNP analysis (Fig 2) when compared to WG-hqSNP analysis (Fig 1) in all three clades possibly because Lyve-SET is very conservative when calling nucleotide variants and as a result, the percentage of called variants reported is usually lower than those from Parsnp. Additionally, unlike Lyve-SET, which uses pre-processed raw sequencing reads as input, Parsnp uses assembled genomes where there are opportunities for errors to occur during assembly that could contribute to the higher SNP count.

Our results show that median number of pairwise hqSNPs in a clonal ETEC O6 outbreak is less than 5 (Table 1). We observed three clusters where isolates from sporadic infections closely clustered with outbreak isolates (Table 2 and Fig 1). Isolates within the first and third clusters are temporally associated so a possible common source cannot be excluded. The other possibility is the existence of common widely distributed sequence types. While the two isolates from Guatemala in the second cluster are geographically and temporally associated, the outbreak related isolate in that cluster was recovered two years later so this case is more likely to represent a common clone. Our study findings seem to be in agreement with the conclusions of von Mentzer *et al* [14] and Sahl *et al* [15] where genetic similarity of ETEC clones were identified in strains isolated from temporally and geographically dispersed cases of ETEC mediated diarrhea. The number of samples is small due to lack of routine ETEC surveillance in the US and the depth of sampling for each outbreak is also small. With a larger sample size, we would expect the within outbreak variability to increase slightly.

Three of the outbreaks were deemed to be polyclonal in nature, i.e., caused by more than one strain of ETEC (Table 1, Figs 1 and 2). The isolates from OB3 were from neighboring states and both cases reported consuming the same cheese product from a local creamery (S1 Table). OB15 and OB18 both took place on cruise ships. The majority of the cruise ship outbreaks investigated in the USA are polyclonal. Typically, the source is a food that is collected from one of the harbors the ship visits to replenish vessel’s food supply. Cruise ships of the outbreaks in this study have mostly sailed on the Caribbean Sea and/or South America. Countries along this cruise route do not have as rigorous food safety standards as the USA, therefore foods can be heavily contaminated with multiple strains of the same species/serotype or even multiple species, which is the definition of a polyclonal outbreak [43]. We also attribute polyclonality as the reason for different resistance profiles of clone 1 and clone 2 isolates of OB15 (Fig 3).

Twenty-two ETEC O6 isolates were resistant to one or more antibiotics out of which 9 were multi-drug resistant (Table 2). This is in line with results from Medina *et al* [11] where ETEC from Peruvian children were also resistant to older inexpensive antibiotics such as ampicillin, trimethoprim-sulfamethoxazole and tetracycline while being susceptible to ciprofloxacin and cephalosporins. Since many AR determinants are on plasmids or other mobile elements [14], there are opportunities for the loss of mobile elements during sub-culturing for testing. In this study, WGS was conducted prior to AST and there were chances for AR determinants to be lost by sub-culturing between sequencing and AST. We attribute this to be the reason for some discrepancies observed between phenotypic AR and resistant determinants in this study. 2015EL1280-1 was phenotypically susceptible to ampicillin (MIC = 2 μg/ml), streptomycin (MIC = 8 μg/ml), sulfisoxazole (MIC < = 16 μg/ml) and trimethoprim-sulfamethoxazole (MIC < = 0.12/2.38 μg/ml) while the corresponding encoding elements were present in these genomes. The resistance determinants were present at 100% nucleotide identity and 100% gene coverage when compared to their best match in ResFinder, but were on a contig with sequencing coverage that was approximately 25% of the average genome coverage (contig coverage 19x; average genome coverage 80x). This observation could be the result of the loss of these resistance determinants in some cells that underwent sequencing and subsequent susceptibility testing. Similarly, 2012EL1408-1 was susceptible to tetracycline (MIC was < = 4 μg/ml) while *tetB* was present in its genome. The *tetB* gene was nearly identical to the reference in ResFinder, with a single amino acid change (P>H) present at amino acid 390 (data not presented). 2013EL1377-9 possessed a *bla*TEM-1B gene in the absence of ampicillin resistance, however upon further examination it was observed that only 63% percent of the gene was present due to an interruption by IS*26* (data not presented), which likely explains the phenotype observed. 2016EL1012-b was sensitive to streptomycin, yet an *aadA1* gene was present in this isolate at 96.46% identity and 100% gene coverage. Closer examination did not reveal any premature stop codons, but did reveal several non-synonymous mutations in the coding sequence (data not presented). The MIC to streptomycin was 16 μg/mL. The US National Antimicrobial Resistance Monitoring System (NARMS) employs an epidemiological cutoff value (ECV) of ≥ 32 μg/mL for streptomycin as there are no standardized breakpoints for Enterobacteriaceae. While most isolates with streptomycin MICs below this ECV do not possess resistance determinants, *E*. *coli* isolates with an *aadA* gene have been encountered with MICs of 8 and 16 μg/mL [44].

Finally, *aad*A1 and *qnrS1* were detected in 2015EL-1279-2 at 100% nucleotide identity and gene coverage in the absence of streptomycin or quinolone resistance (Table 2). As noted previously, while most isolates with streptomycin MICs below the ECV for this drug do not possess resistance determinants, *E*. *coli* isolates with an *aadA* gene have been encountered with lower MIC values [44]. Plasmid-mediated quinolone resistance genes (PMQR) such as the *qnr* gene has been observed to confer low level fluoroquinolone resistance (i.e. ciprofloxacin), with little impact on nalidixic acid MICs[31]. PMQR genes in association with chromosomal resistance mechanisms significantly increase the resistance to quinolones and fluoroquinolones. The decreased susceptibility conferred by *qnr* genes is generally not sufficient to raise the MIC over the current CLSI upper limit of the susceptible range (≤ 1 μg/mL for *E*. *coli* and *Shigella*.*)* [34]. The MIC for ciprofloxacin in 2015EL-1279-2 was 0.25 μg/mL which is in the expected range for *qnr*+ *E*. *coli* [45] and this genome did not harbor chromosomal mutations to *gyrA* and/or *parC* genes in the QRDR.

In conclusion, ETEC O6 isolates in this study showed significant genomic diversity [46] by phylogenetic analyses independent of time and geographical location of isolation. However, a few clonal subtypes causative of an outbreak and/or sporadic infections were observed. The phylogenetic relationships between the isolates were similar by WG-hqSNP and CG SNP analyses thereby providing support that they represent true evolutionary relationships. Based on our results, fewer than 5 pairwise hqSNPs between ETEC O6 isolates is circumstantial evidence of an outbreak cluster. This study provides a baseline for understanding the ETEC O6 population structure by WG-hqSNP and CG-SNP analyses as PulseNet USA moves forward with implementation of routine surveillance for ETEC using WGS. Our study identified multi-drug resistance among 6 of 27 outbreak isolates and 3 of 13 sporadic isolates, a finding that is concerning and warrants further investigation. Similar genome wide characterization studies of other dominant circulating serogroups of ETEC and/or other non Shiga toxin producing *E*. *coli* such as enteropathogenic *E*. *coli* (EPEC), enteroaggregative *E*. *coli* (EAEC) and diffuse-adhering enteroinvasive *E*. *coli* (EIEC) are needed to understand their population structure and AR patterns.

## Supporting information

S1 Fig ETEC O6 isolates are clustered into four clades by WG-hqSNP analysis. Whole genome high-quality single nucleotide polymorphisms (WG-hqSNP) analysis tree generated by Lyve-SET for phylogenetic relatedness of 40 ETEC O6 isolates against the reference genome 2011EL1370-2. The scale represents a distance of 0.002 hqSNPs per site. At each ancestor node, bootstrap percentages are displayed. Isolates clustered into 4 major clades. Isolates are clustered into Clade I, Clade II and Clade III. Isolates are color coded based on isolation during an outbreak (OB) or sporadic infection (S). In the metadata table, *CS stands for Cruise Ship; US states are abbreviated.
(TIF)

S1 Table Bacterial isolates used in this study. (DOCX)
