# Supplementary figures and images for: Genome wide characterization of enterotoxigenic Escherichia coli serogroup O6 isolates from multiple outbreaks and sporadic infections from 1975-2016
Source: PLoS One. 2018 Dec 31;13(12):e0208735. doi: 10.1371/journal.pone.0208735 (PMC6312315; doi:10.1371/journal.pone.0208735)

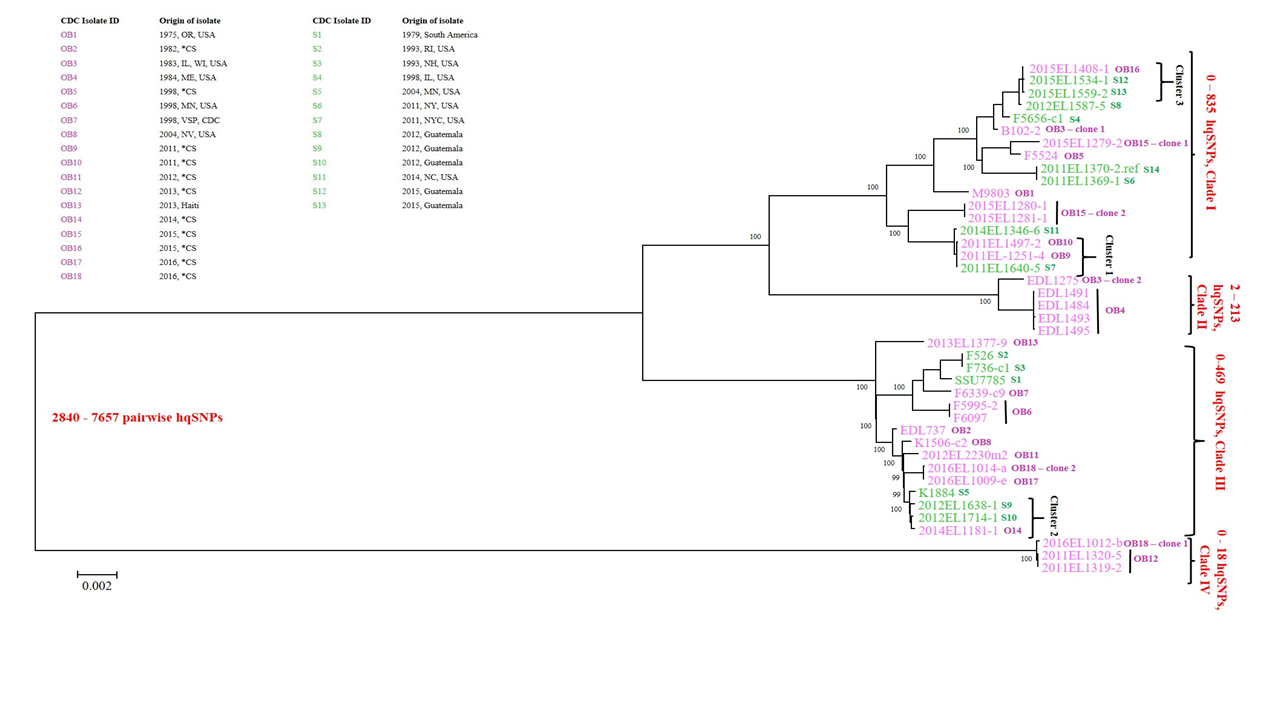

Supplement: S1 Fig — Whole genome high-quality single nucleotide polymorphisms (WG-hqSNP) analysis tree generated by Lyve-SET for phylogenetic relatedness of 40 ETEC O6 isolates against the reference genome 2011EL1370-2. The scale represents a distance of 0.002 hqSNPs per site. At each ancestor node, bootstrap percentages are displayed. Isolates clustered into 4 major clades. Isolates are clustered into Clade I, Clade II and Clade III. Isolates are color coded based on isolation during an outbreak (OB) or sporadic infection (S). In the metadata table, *CS stands for Cruise Ship; US states are abbreviated. (TIF) [file pone.0208735.s001.tif]
