# Supplementary material for: Genome wide characterization of enterotoxigenic Escherichia coli serogroup O6 isolates from multiple outbreaks and sporadic infections from 1975-2016
Source: PLoS One. 2018 Dec 31;13(12):e0208735. doi: 10.1371/journal.pone.0208735 (PMC6312315; doi:10.1371/journal.pone.0208735)
Supplement: S1 Table — (DOCX) [file pone.0208735.s002.docx]

**S1 Table: Bacterial isolates used in this study**

| CDC isolate identification (ID) | O:H serotype | NCBI Accession Number | Origin of isolate  (Year and location of isolation) | Outbreak (OB)/Sporadic (S) infection number | Virulence Factors |
| --- | --- | --- | --- | --- | --- |
| M9803 | O6:H16 | SAMN07702527 | 1975, OR, USA | OB1 | *ltcA, astA* |
| EDL737 | O6:H16 | SAMN07702220 | 1982, *CS | OB2 | *ltcA, astA* |
| B102-2 | O6:H16 | SAMN07702525 | 1983, IL, USA | OB3 | *ltcA, astA* |
| EDL1275 | O6:H16 | SAMN07702225 | 1983, WI, USA | OB3 | *ltcA, astA* |
| EDL1491 | O6:H16 | SAMN07702223 | 1984, ME, USA | OB4 | *ltcA, astA* |
| EDL1493 | O6:H16 | SAMN07702222 | 1984, ME, USA | OB4 | *ltcA* |
| EDL1484 | O6:H16 | SAMN07702224 | 1984, ME, USA | OB4 | *ltcA, astA* |
| EDL1495 | O6:H16 | SAMN07702221 | 1984, ME, USA | OB4 | *ltcA, astA* |
| F5524 | O6:H16 | SAMN07702526 | 1998, Oceanbreeze, *CS | OB5 | *ltcA, astA* |
| F5995 | O6:H16 | SAMN07702524 | 1998, MN, USA | OB6 | *ltcA, astA* |
| F6097 | O6:H16 | SAMN07702523 | 1998, MN, USA | OB6 | *ltcA, astA* |
| F6339-c9 | O6:H16 | SAMN07702522 | 1998, Vessel Sanitation Program, CDC | OB7 | *ltcA, astA* |
| K1506-c2 | O6:H16 | SAMN07702535 | 2004, NV, USA | OB8 | *ltcA, astA* |
| 2011EL1251-4 | O6:H16 | SAMN07702534 | 2011, Oceania, *CS | OB9 | *ltcA, astA* |
| 2011EL1497-2 | O6:H16 | SAMN07702533 | 2011, *CS | OB10 | *ltcA, astA* |
| 2012EL2230m2 | O6:H16 | SAMN04578492 | 2012, *CS | OB11 | *ltcA, astA* |
| 2013EL1319-2 | O6:H16 | SAMN02863978 | 2013, Veendam, *CS | OB12 | *astA* |
| 2013EL1320-5 | O6:H16 | SAMN02851527 | 2013, Veendam, *CS | OB12 | *astA* |
| 2013EL1377-9 | O6:H16 | SAMN07702212 | 2013, Laboratoire National de Sante Publique (LNSP), Haiti | OB13 | *ltcA, astA* |
| 2014EL1181-1 | O6:H16 | SAMN03333592 | 2014, Zuiderdam, *CS | OB14 | *ltcA, astA* |
| 2015EL1279-2 | O6:H16 | SAMN04578477 | 2015, Holland America, *CS | OB15 | *ltcA, astA* |
| 2015EL1280-1 | O6:H16 | SAMN07702213 | 2015, Holland America, *CS | OB15 | *ltcA* |
| 2015EL1281-1 | O6:H16 | SAMN07702218 | 2015, Holland America, *CS | OB15 | *ltcA* |
| 2015EL1408-1 | O6:H16 | SAMN04578537 | 2015, Oceania, *CS | OB16 | *ltcA, astA* |
| 2016EL1009-e | O6:H16 | SAMN07702216 | 2016, Oceania Regatta, *CS | OB17 | *astA* |
| 2016EL1012-b | O6:H16 | SAMN07702227 | 2016, Ocean Princess, *CS | OB18 | *astA* |
| 2016EL1014-a | O6:H16 | SAMN07702217 | 2016, Ocean Princess, *CS | OB18 | *ltcA, astA* |
| SSU7785 | O6:H16 | SAMN07702219 | 1979, El Salvador, South America | S1 | *astA* |
| F526 | O6:NM | SAMN07702532 | 1993, RI, USA | S2 | *ltcA, astA* |
| F736-c1 | O6:NM | SAMN07702531 | 1993, NH, USA | S3 | *ltcA, astA* |
| F5656-c1 | O6:H16 | SAMN04621488 | 1998, IL, USA | S4 | *ltcA, astA* |
| K1884 | O6:H16 | SAMN07702530 | 2004, MN, USA | S5 | *ltcA, astA* |
| 2011EL1369-1 | O6:H16 | SAMN07702529 | 2011, NY, USA | S6 | *ltcA, astA* |
| 2011EL1640-5 | O6:H16 | SAMN07702528 | 2011, NYC, USA | S7 | *ltcA, astA* |
| 2012EL1587-5 | O6:H16 | SAMN07702451 | 2012, Guatemala | S8 | *ltcA, astA* |
| 2012EL1638-1 | O6:H16 | SAMN07702450 | 2012, Guatemala | S9 | *ltcA, astA* |
| 2012EL1714-1 | O6:H16 | SAMN07702449 | 2012, Guatemala | S10 | *ltcA, astA* |
| 2014EL1346-6 | O6:H16 | SAMN07702226 | 2014, NC, USA | S11 | *ltcA, astA* |
| 2015EL1534-1 | O6:H16 | SAMN07702215 | 2015, Guatemala | S12 | *ltcA, astA* |
| 2015EL1559-2 | O6:H16 | SAMN07702214 | 2015, Guatemala | S13 | *ltcA, astA* |
| *2011EL1370-2  (Reference isolate) | O6:H16 | CP022912, CP022913, CP022914 | 2011, NY, USA | S14 | *ltcA, astA* |

### Footnote: All isolates were sequenced by 500 cycle chemistry in MiSeq except 2012EL2230m2, 2015EL1279-2 and 2015EL1408-1, which were sequenced by HiSeq 500 cycle chemistry. *CS is Cruise Ship; *2011EL1370-2 (GCF_002269325.1) is the reference genome used for WG-hqSNP and SNP analyses. 2016EL1012-b, 2011EL1320-5 and 2011EL1319-2 were highly divergent from clusters I, II, and III.

### VirulenceFinder 1.5 prediction: Heat-labile enterotoxin A subunit *(ltcA)* and heat-stable enterotoxin 1 *(astA)*.
